# Effect of out-of-village working activities on recent malaria exposure in the Peruvian Amazon using parametric g-formula

**DOI:** 10.1038/s41598-022-23528-8

**Published:** 2022-11-09

**Authors:** Gabriel Carrasco-Escobar, Jason Rosado, Oscar Nolasco, Michael T. White, Ivo Mueller, Marcia C. Castro, Hugo Rodriguez-Ferruci, Dionicia Gamboa, Alejandro Llanos-Cuentas, Joseph M. Vinetz, Tarik Benmarhnia

**Affiliations:** 1 Herbert Wertheim School of Public Health and Human Longevity Science, University of California San Diego, La Jolla, CA USA; 2 Health Innovation Lab, Institute of Tropical Medicine “Alexander Von Humboldt”, Universidad Peruana Cayetano Heredia, Lima, Peru; 3 G5 Épidémiologie Et Analyse Des Maladies Infectieuses, Département de Santé Globale, Institut Pasteur, 75015 Paris, France; 4 Instituto de Medicina Tropical Alexander Von Humboldt, Universidad Peruana Cayetano Heredia, Lima, Peru; 5 Laboratorio ICEMR-Amazonia, Laboratorios de Investigación Y Desarrollo, Facultad de Ciencias Y Filosofía, Universidad Peruana Cayetano Heredia, Lima, Peru; 6 Department of Medical Biology, University of Melbourne, Melbourne, Victoria Australia; 7 Population Health and Immunity Division, Walter and Eliza Hall Institute of Medical Research, Melbourne, Australia; 8 Department of Global Health and Population, Harvard T.H. Chan School of Public Health, Boston, MA USA; 9 Universidad Nacional de La Amazonía Peruana, Loreto, Peru; 10 Departamento de Ciencias Celulares Y Moleculares, Facultad de Ciencias Y Filosofía, Universidad Peruana Cayetano Heredia, Lima, Peru; 11 Section of Infectious Diseases, Department of Internal Medicine, Yale School of Medicine, New Haven, CT USA; 12 Scripps Institution of Oceanography, University of California, San Diego, CA 92037 USA

**Keywords:** Malaria, Epidemiology

## Abstract

In the Amazon Region of Peru, occupational activities are important drivers of human mobility and may increase the individual risk of being infected while contributing to increasing malaria community-level transmission. Even though out-of-village working activities and other mobility patterns have been identified as determinants of malaria transmission, no studies have quantified the effect of out-of-village working activities on recent malaria exposure and proposed plausible intervention scenarios. Using two population-based cross-sectional studies in the Loreto Department in Peru, and the parametric g-formula method, we simulated various hypothetical scenarios intervening in out-of-village working activities to reflect their potential health benefits. This study estimated that the standardized mean outcome (malaria seroprevalence) in the unexposed population (no out-of-village workers) was 44.6% (95% CI: 41.7%–47.5%) and 66.7% (95% CI: 61.6%–71.8%) in the exposed population resulting in a risk difference of 22.1% (95% CI: 16.3%–27.9%). However, heterogeneous patterns in the effects of interest were observed between peri-urban and rural areas (Cochran’s *Q* test = 15.5, *p* < 0.001). Heterogeneous patterns were also observed in scenarios of increased prevalence of out-of-village working activities and restriction scenarios by gender (male vs. female) and age (18 and under vs. 19 and older) that inform possible occupational interventions targetting population subgroups. The findings of this study support the hypothesis that targeting out-of-village workers will considerably benefit current malaria elimination strategies in the Amazon Region. Particularly, males and adult populations that carried out out-of-village working activities in rural areas contribute the most to the malaria seropositivity (recent exposure to the parasite) in the Peruvian Amazon.

## Introduction

The Amazon rainforest located in the World Health Organization (WHO) Region of the Americas remains a malaria hotspot. Within the 19 countries in this Region, more than 600,000 (presumed and confirmed) incident cases were estimated in 2020^1^ and 9 countries shared the Amazonian territory and most of the malaria cases: Bolivia, Brazil, Colombia, Ecuador, French Guiana (France), Guyana, Suriname, Venezuela, and Peru^1^. Malaria transmission in this area is dominated by *Plasmodium vivax* (75%) followed by *P. falciparum* and mixed (25%) infections. Most of these cases are located within 14 subnational units only^1^ which include the Loreto Region located in the Peruvian Amazon. Historically, 93.1% of cases in Peru were reported in this Region^2^ that are mainly transmitted by *Nyssorinchus* (*Anopheles) darlingi*^3,4^.

In the last two decades, many interventions aiming at reducing the incidence of malaria in Peru have been implemented. For example, the PAMAFRO project (2005–2010)^5^ focused on training community health workers for early diagnosis, monitoring, and treatment of malaria, the use of long-lasting insecticide-treated nets (LLINs), and community education in malaria prevention measures. Shortly after the interruption of the PAMAFRO project, the “Plan Malaria Cero” (PMC; 2017–2021)^6^ was implemented with the aim to eliminate malaria transmission in three stages over a 25-year timeframe. More recently, a new “Plan Hacia la Eliminación de la Malaria en el Perú” (2022–2030)^7^ aims to provide the legal, economic, and political support to achieve malaria elimination in Peru by applying a set of evidence-based interventions. However, while most of these activities contributed to a reduction of malaria incident cases^8^, many dimensions regarding malaria transmission, such as human mobility, are still not considered and constitute missed opportunities for alternative interventions to ultimately eliminate malaria. The WHO guidelines for elimination and prevention of reintroduction strategies recently highlighted the important role of human mobility as a challenge for sustaining malaria elimination efforts^9,10^. Human mobility was described as an associated factor for both malaria exposure^11^ and infection^12^ in the Amazon Region.

In the Amazon Region of Peru, an important driver of human mobility is related to occupational activities^13,14^. Indeed, many workers engage in out-of-village working activities in order to meet job opportunities and thus may increase their risk of being infected but also may contribute to increasing community-level transmission^12,14^. Previous studies have identified occupational mobility as a determinant for malaria risk and used forest goers^15–17^ or out-of-village activities^12,18^ as measures of such exposure but did not rely on causal modelling nor objectively measured exposures and malaria outcomes. Furthermore, no study simulated plausible scenarios to assess the potential benefits of hypothetical interventions.

The contribution of occupational determinants of infectious diseases has received more attention recently in the context of the COVID-19 pandemic^19^ but evidence about which strategies targeting occupational mobility may be most effective at reducing malaria risk is lacking. Therefore, simulating the potential benefits of various intervention scenarios could be particularly helpful to design future occupational interventions to complement already implemented community-based actions to ultimately reach malaria elimination. Some modern causal inference methods, including the parametric g-formula, have been proposed to flexibly estimate the effect of different exposure regimes. Parametric g-formula methods are a generalization of standardization methods that can simulate different hypothetical interventions on the exposure of interest^20^. While many studies have recently relied on such an approach, including in occupational settings^21–23^, to simulate hypothetical interventions, such methods have been applied to a limited extent in the context of malaria epidemiology^24,25^.

In addition, many population characteristics may modulate the effect of out-of-village activities on malaria risk and may inform targeted interventions. First, individual-level characteristics such as gender and age may constitute important effect modifiers. Yet, cultural, geographical, and social characteristics may also greatly differ between rural and peri-urban areas in the Peruvian Amazon. Previous studies found contrasting differences in the proportion of inhabitants that participate in out-of-village activities between rural and peri-urban areas in Iquitos^11^. Also, contrasting patterns were reported in malaria infection rate^12,18^, seropositivity (exposure to previous infection)^11^, and parasite genetic population structure^26,27^ between these areas. In regions with rural-to-urban gradients, ecological factors increase disparities in malaria susceptibility^28^ driven by marked variations in *Ny. darlingi* abundance and biting behavior across the forest, chacra (crop fields) (perturbed secondary forest), and urban settings^29,30^.

Thus, this study aims to estimate the effect of out-of-village mobility on malaria exposure in contrasting geographic areas to better inform occupational interventions related to malaria elimination strategies in the Amazon Region. Using two population-based cross-sectional studies in the Loreto Department in Peru, and g-formula methods, we simulated various hypothetical scenarios intervening in out-of-village working activities and various population subgroups to reflect the potential health benefits of future interventions.

## Methods

### Ethics

This study analyzed data from two studies that were approved by the Ethics Review Board of the Regional Health Directorate of Loreto and Universidad Peruana Cayetano Heredia in Lima: the Circles of Research on Arboviruses and Malaria (CAM) study (SIDISI 101645/2017) and the Amazonia International Center of Excellence in Malaria Research (ICEMR) study (SIDISI 101518/2018). Participants in both studies were enrolled upon signing an informed consent or informed assent in case of participants under 18 years old. All the methods were carried out in accordance with the approved guidelines.

### Study design

We conducted etiological and simulation studies to quantify the role of out-of-village working activities on recent malaria exposure in two population-based cross-sectional studies carried out in the Loreto Department, Peru. The designs of both studies were described elsewhere^31^. Briefly, both studies were conducted by the same research team in different months in 2018. A structured questionnaire, georeferencing of households, and blood samples were collected in 10 villages in two districts of Loreto: Iquitos—mostly urban—in April 2018, and Mazán—mostly rural—in July 2018. Here, the—previously reported^31^—seropositivity status of the participants (based on a random forest classifier) was used in combination with a parametric g-formula (see details below) to compute the average causal effect of out-of-village mobility on malaria exposure. In addition, we simulated multiple scenarios of mobility restrictions (by proportion of travelers, gender, and age) to estimate the impact of such restriction policies in reducing malaria exposure in the Peruvian Amazon.

### Study site and population

**Figure 1:**
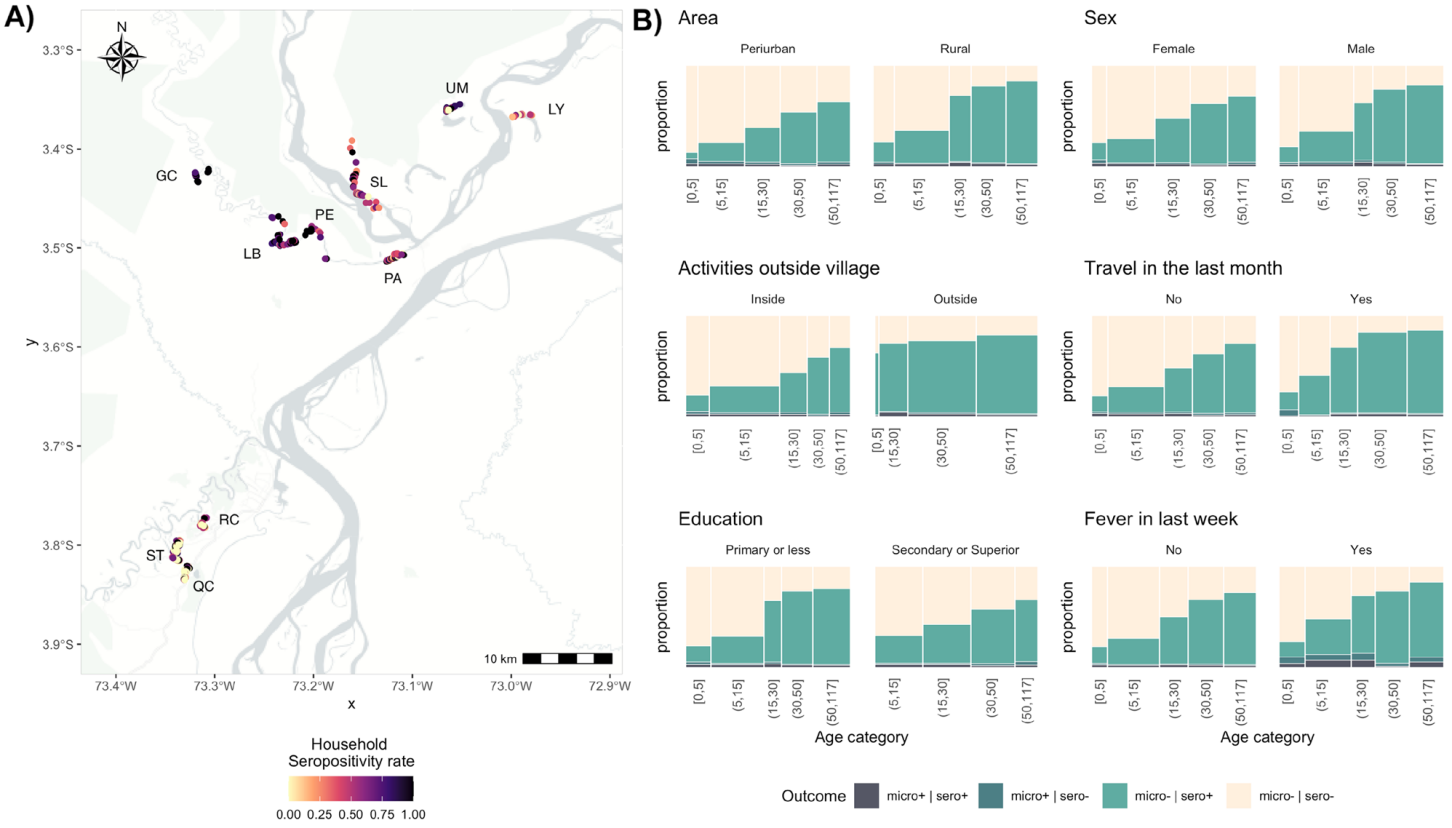
Study area and socio-demographic distribution of seropositivity in the Loreto department in the Peruvian Amazon. (**A**) Seropositivity rate at household level in the villages of Iquitos district (bottom left): Rumococha (RC), Santo Tomas (ST), Quistococha (QC), and Mazán district (top right): Gamitanacocha (GC), Libertad (LB), Primero de Enero (PE), Puerto Alegre (PA), Salvador (SL), Lago Yuracyacu (LY), and Urco Miraño (UM). (**B**) Distribution of serology (sero) and microscopy (micro) rates across age categories and sociodemographic variables. Maps were produced using R v.4.1 (R Development Core Team, R Foundation for Statistical Computing, Australia) based on public geographic data extracted from OpenStreetMap contributors (www.openstreetmap.org) under Open Data Commons Open Database License (ODbL) 1.0 (http://openstreetmap.org/copyright).

High-risk malaria villages were selected in peri-urban and rural areas based on Ministry of Health (MoH) historical data (Fig. 1A). Three villages were selected in the peri-urban area: Rumococha (RM), Santo Tomás (ST), and Quistococha (QC). These villages are located on the outskirts of Iquitos district, 10 km from Iquitos City (capital of Loreto; lat: 03°44.591 ′S, long: 73°19.615 ′W), accessible by road and highly deforested. Seven villages were selected in the rural area: Gamitanacocha (GC), Libertad (LB), Primero de Enero (PE), Puerto Alegre (PA), Salvador (SL), Lago Yuracyacu (LY), and Urco Miraño (UM). These villages are located in the Mazán district, accessible only by boat (~ 2–7 h from Iquitos city) and characterized by dense primary and secondary forest cover. All participants 6 months or older at the date of survey were invited to the study if they lived in the selected village and gave consent to donate a blood sample by venipuncture for malaria diagnosis.

### Data collection and variable definitions

A full census of the study populations was conducted in April-July 2018. Individual and household data on socio-demographics (age, gender, education, occupation), self-reported previous history of clinical malaria, and structural characteristics of the household were collected. All households and participants were encoded and geo-referenced using a Global Positioning System (GPS) handheld device (Garmin’s GPSMAP 60CSx, Garmin International Inc., USA).

A blood sample of 6 mL for adults or 3 mL for children of whole blood was collected by venipuncture in tubes with EDTA (BD Vacutainer, BD Franklin Lakes, USA) as a preservative. Venipuncture blood samples were separated by centrifugation (3500 rpm) into plasma and packed red blood cells (PRBC) for serological analysis.

The primary exposure–out-of-village working activities–and covariates were collected in structured questionaries. All villagers self-reported whether they traveled in the previous month (travel history), sex, age, and occupation. All occupational activities were grouped into a binary variable according to the location where the activities were carried out (inside or outside their home village). Previous studies identified that out-of-village working activities in these areas include logging, hunting, fishing, trading, and farming^12,13,32^.

The primary outcome–malaria serological exposure– was defined according to a serological assay that target *Plasmodium* species-specific levels (recent infection up to 9 months in the past)^33^. IgG antibody responses to 8 serological exposure markers (SEM) to *P. vivax* were measured using a Luminex® platform, as described elsewhere^34^. The 8 SEM panel has been previously validated^33^ and consisted of the following proteins: PVX_099980 (19 kDa C-terminal region of merozoite surface protein 1, PvMSP119), PVX_096995 (tryptophan-rich antigen, Pv-fam-a, PvTRAg_2), PVX_112670 (PvTRAg_28), PVX_097625 (merozoite surface protein 8, putative, PvMSP8), PVX_097720 (merozoite surface protein 3, PvMSP3.10), PVX_087885 (rhoptry-associated membrane antigen, putative, PvRAMA), PVX_094255 (reticulocyte binding protein 2b, PvRBP2b) and KMZ83376.1d (erythrocyte-binding protein II, PvEBPII). To normalize and diminish inter-plate variation, a standard curve was prepared using a plasma pool of hyper-immune adults from Papua New Guinea. Relative Antibody Units (RAU) or dilutions were obtained by extrapolating the Median Fluorescence Intensity (MFI) in a standard curve by a 5 parameters logistic model. Seropositivity to each marker was defined by using a Random Forests based classification algorithm previously validated in low *P. vivax* transmission contexts^33^. Further description of the serological makers and the measured structure of the transmission in the area could be found elsewhere^31^.

### Estimating the average causal effect of out-of-village working activities on malaria

To estimate the average causal effect of out-of-village working activities on malaria a parametric g-computation described previously^20,23,35–38^ was used. G-formula (also known as g-computation) can be seen as a generalization of standardization methods applied to multiple settings and first described in 1986^39^. In the g-formula, under identification assumptions such as exchangeability, consistency and positivity conditional on the variables in *L* (potential confounders), the standardized mean outcome is the weighted average of the conditional means using as weights the prevalence of each stratum *l* of the vector of confounders *L* in the study population computed as follows:

$$\mathop \sum \limits_{l} E\left[ {Y{|}A = a,L = l} \right] \times Pr\left[ {L = l} \right]$$

where $E[Y|A = a,L = l]$ are the conditional means in each of the strata *l* and $Pr\left[ {L = l} \right]$ is the prevalence of *l*. Such quantities are estimated parametrically. The following 4-step process was adopted. First, expansion of the original dataset; a new set of analytic datasets was created by repeating the original dataset in three blocks. The first block was identical to the original dataset, the second block was modified and set the values of A (of out-of-village working activities) to unexposed (*A* = *0*), the third block was modified and set the values of A to exposed (*A* = *1*). In the second and third blocks, the values of the outcome (*Y* – malaria exposure) were removed and set as missing. Second, a regression model (a modified Poisson regression^40^ to consider the highly prevalent outcome) was fitted for the outcome (i.e. malaria) given exposure *A* (out-of-village working activities) and confounders *L* (including villages as fixed effects). The variables used for the model estimation were age, sex, education, and fever history. The final model included interactions between the main exposure and age and sex. It is worth mentioning that only data in the first block contributed to the estimation (as Y was absent from the created blocks). Third, the parameters estimated using data from the first block were used to predict the outcome values for all observations in the second and third blocks, which standardizes based on the empirical distribution of confounders. The average of all predicted values in the second and third block is precisely the standardized mean outcome in the unexposed and exposed, respectively. Finally, risk differences and ratios can be estimated by comparing such estimated counterfactual quantities. To obtain 95% Confidence Intervals (CI), a Monte Carlo resample with 999 replicates was drawn with replacement from the original data. These analyses were further explored by stratifying by age, proportion of travelers, and gender as well as location in peri-urban or rural settings.

### Simulation of restriction scenarios

We then conducted a series of simulations to (synthetically) modify the prevalence of the main exposure (out-of-village working activities) while keeping the confounding structure, to explore scenarios where the main exposure would vary and compared to the natural course (i.e., the initial/observed setting or said differently, in the absence of any interventions) to inform future interventions. We tested scenarios of the prevalence of out-of-village working activities ranging from 0 to 1 through incremental steps of 0.1. We stratified our analyses by peri-urban or rural settings. In addition, 4 scenarios were tested based on full (FE) and null (NE) exposure in relation to gender (male vs. female) and age (18 and under vs. 19 and older) to inform possible occupational interventions targetting population subgroups.

## Results

### Baseline characteristics

A total of 785 individuals from 421 households were enrolled in the Iquitos district (peri-urban setting) and 1005 individuals from 419 households in the Mazán district (rural setting). The village sample size range between 250 and 273 individuals in peri-urban settings and between 47 and 270 individuals in rural areas. The average age of the population was 27.7 (SD = 22.2) years in the rural area and 30.6 (SD = 21.8) years in the peri-urban area. Important differences were observed in the proportion of females (59% vs 5.1%), secondary or superior education (46% vs. 27%), work inside the village (88% vs. 59%), and travel in the last month (2% vs. 33%) between peri-urban and rural settings.

### Malaria seroprevalence rate

An overall seroprevalence rate of 49% was observed in the study population. However important differences were observed across settings (Table 1). A higher seroprevalence was observed in rural areas (57%) in comparison to peri-urban areas (39%). Importantly, a seroprevalence rate higher than 40% was observed in 6 (GC, LB, PE, SL, PA, and UM) out of the 7 rural villages and only in 1 (ST) out of the 3 peri-urban villages. The highest seroprevalences were observed in GC (87%), LB (75%), and PE (69%), all located in the rural district of Mazán. On average, the age of seropositive participants is higher (37 years old) in comparison to seronegative participants (21 years old). Slight differences in the seropositivity status were observed in relation to gender and education, however, contrasting patterns were observed in relation to outside (77%) in comparison to inside (38%) workers and recent travelers (66%) in comparison to no travelers (45%).

**Table 1:** Baseline characteristics of the study population and their malaria seropositive status.

	Negative (N = 917)	Positive (N = 873)	Overall (N = 1790)
**Area**			
Peri-urban	481 (52.5%)	304 (34.8%)	785 (43.9%)
Rural	436 (47.5%)	569 (65.2%)	1005 (56.1%)
**Age**			
[0,5]	147 (16.0%)	31 (3.6%)	178 (9.9%)
[5, 15]	405 (44.2%)	163 (18.7%)	568 (31.7%)
[15, 30]	148 (16.1%)	151 (17.3%)	299 (16.7%)
[30, 50]	126 (13.7%)	262 (30.0%)	388 (21.7%)
[50,117]	91 (9.9%)	266 (30.5%)	357 (19.9%)
**Gender**			
Female	536 (58.5%)	437 (50.1%)	973 (54.4%)
Male	381 (41.5%)	436 (49.9%)	817 (45.6%)
**Education**			
Primary or less	582 (63.5%)	601 (68.8%)	1183 (66.1%)
Secondary or superior	335 (33.9%)	272 (31.2%)	607 (33.9%)
**Work type**			
Inside village	798 (87.0%)	480 (55.0%)	1278 (71.4%)
Outside village	119 (13.0%)	393 (45.0%)	512 (28.6%)
Travel in the last month*			
No	794 (86.6%)	642 (73.5%)	1436 (80.2%)
Yes	120 (13.1%)	229 (26.2%)	349 (19.5%)
Microscopy result*			
Negative	894 (97.5%)	835 (95.6%)	1729 (96.6%)
Positive	12 (1.3%)	26 (3.0%)	38 (2.1%)
**Fever**			
No	900 (98.1%)	864 (99.0%)	1764 (98.5%)
Yes	17 (1.9%)	9 (1.0%)	26 (1.5%)

*variable with missing data.

The spatial distribution of the seropositivity rates is shown in Fig. 1A and Supplementary Fig. 1. A clustered pattern at the household level was observed in both study settings. Out of the 421 households surveyed in the peri-urban area, a seropositive individual was detected in 290 (68%) households, ranging from 55 to 82% at the village level. Conversely, in the rural setting, a seropositive individual was detected in 396 (94%) out of 419 households. Remarkably, at least one seropositive participant was detected in all households in GC and PE.

The seroprevalence rates were further explored across age categories and different socioeconomic variables in Fig. 1B. In addition to the overall higher malaria exposure (seroprevalence) in rural than peri-urban areas, the age breakdown showed contrasting patterns between these areas. A smoother increase in the age-seroprevalence trend was observed in peri-urban areas. In contrast, an abrupt disruption at age 15 was observed in rural areas. It is important to notice that the age composition is different between rural/peri-urban areas (distinguished by the width of the bars), much younger in rural areas. Most noticeable differences in the age-structure and age-seroprevalence trend were observed between type of activities (inside/outside village) and recent travelers. Out-of-village working activities were carried out by the older population (> 30 years old) that showed high seroprevalence rates (> 60%). A similar pattern was observed for recent travelers, most of them were adults (> 30 years old) with very high seroprevalence rates (> 80%). The frequency distribution of out-of-village working activities and seropositivity status is shown in Supplementary Fig. 2 and spatial distribution of household work out-of-village rate spatial distribution in the villages in the study area is shown in Supplementary Fig. 3.

### Average causal effect estimation

**Figure 2:**
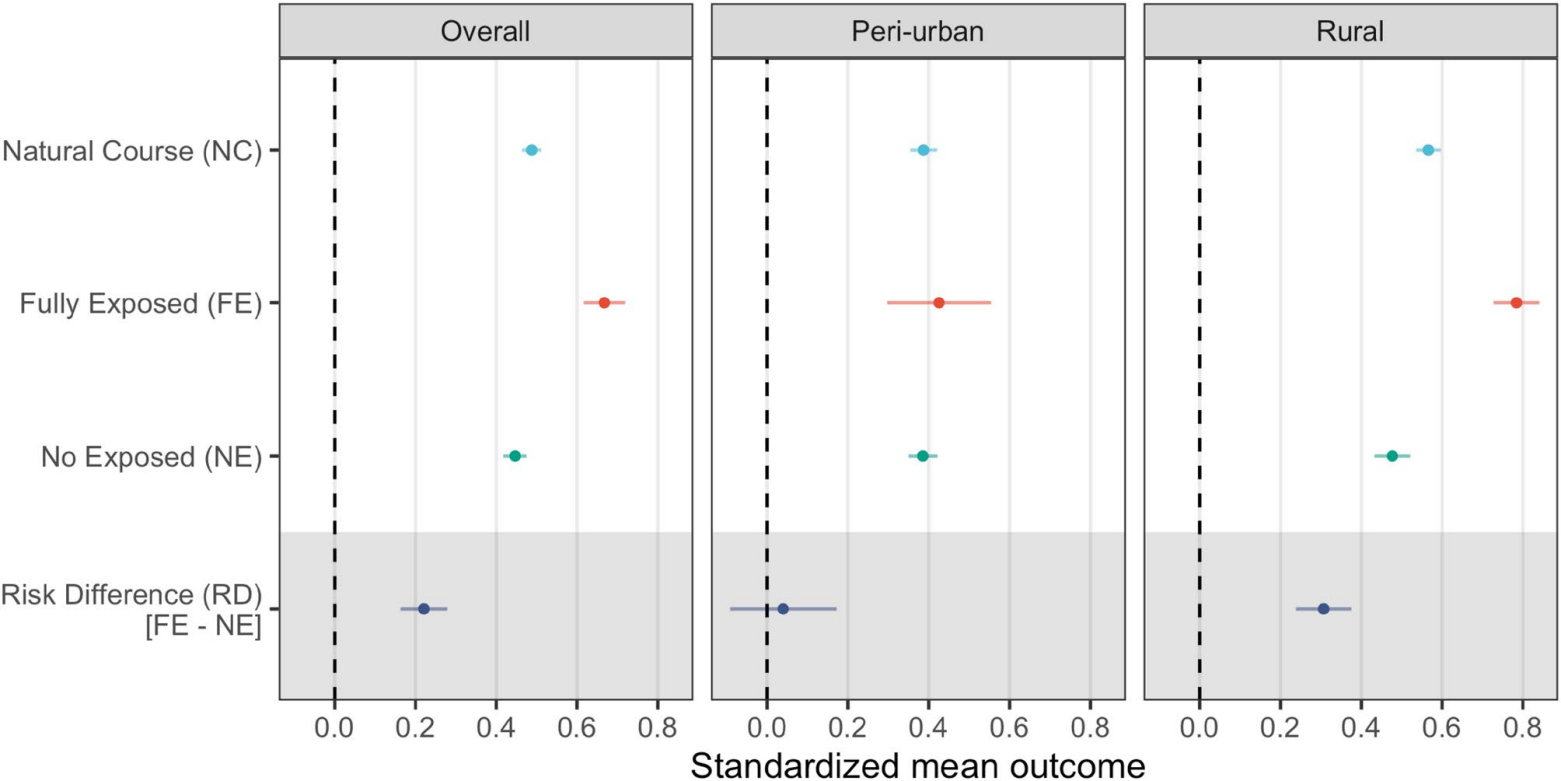
Summary of g-computation estimates by geographic area in the Loreto department in the Peruvian Amazon. Standardized mean outcome estimations for the natural course (NC) and simulated scenarios of full exposure (FE), no exposure (NE) and their risk differences (RD, grey area).

Using a parametric g-computation, this study estimated that the standardized mean outcome (malaria seroprevalence) in the unexposed population (i.e. if all participants do not carry out out-of-village working activities) is 44.6% (95% CI: 41.7%–47.5%) and 66.7% (95% CI: 61.6%–71.8%) in the exposed population (i.e. if all participants carry out out-of-village working activities) (Fig. 2). The role of out-of-village working activities on recent malaria exposure (seroprevalence) was estimated as the difference between these quantities (standardized mean outcome) in the exposed and unexposed. This results in an important and precise average causal effect with a risk difference of 22.1% (95% CI: 16.3%–27.9%).

However, contrasting patterns in standardized mean outcomes and the average causal effects were observed between peri-urban and rural areas. In peri-urban settings, similar standardized mean outcomes were estimated among exposed (42.5%; 95% CI: 29.7%–55.4%) and unexposed (38.6%; 95% CI: 35.0%–42.1%) resulting in no detected differences in malaria risk associated with out-of-village working activities. In the rural areas, the standardized mean outcomes among exposed was 78.4% (95% CI: 72.7%–84.1%) and 47.7% (95% CI: 43.2%–52.1%) among unexposed, resulting in an average causal effect of 30.7% (95% CI: 23.8%–37.6%). Significant heterogeneity in the average causal effect was observed between peri-urban and rural areas (Cochrans *Q* test = 15.5, *p* < 0.001).

### Restriction scenarios

**Figure 3:**
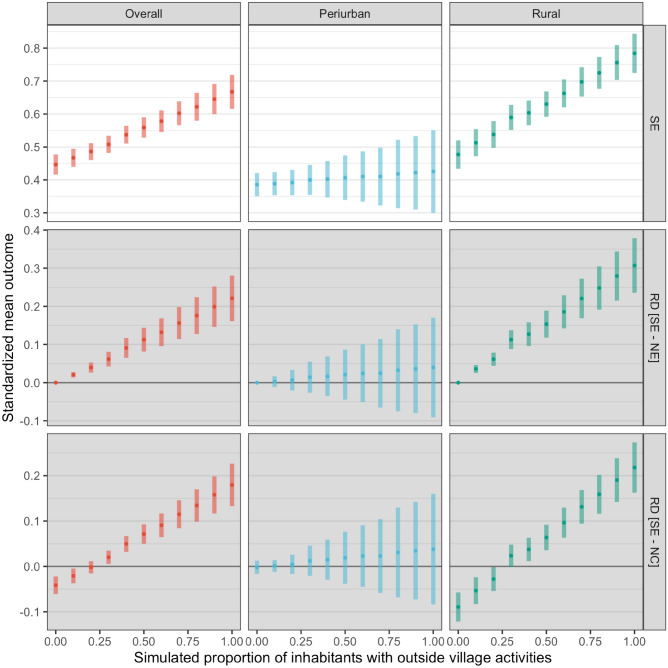
Standardized mean outcome estimated under simulated scenarios by proportion of travelers and geographic area in the Loreto department in the Peruvian Amazon. Standardized mean outcome estimations for the simulated exposure (SE; level of out-of-village working activities) and its risk difference (RD, grey panels) in comparison to no exposure (NE) and the natural course (NC).

Multiple scenarios were tested to inform policy making by simulating the prevalence of out-of-village working activities. Overall, the observed prevalence of out-of-village working activities was 28.6% (Table 1) and the estimated standardized mean outcome (malaria seroprevalence) was 48.8% (95% CI: 46.4%–51.1%) (Fig. 2). After manipulating (by simulation) the prevalence of the exposure (herein referred to as simulated exposure–SE) and computing the corresponding standardized mean outcome, a dose–response curve was constructed for overall, peri-urban, and rural areas (Fig. 3). The average causal effect is–in consequence–the difference between the standardized mean outcome at both extremes of these dose–response curves (0% exposed vs. 100% exposed). Further explorations were conducted by comparing the SE against no (0%) exposure (NE) and the natural course (NC) in each geographic area (Fig. 3). The main role of out-of-village working activities on malaria seroprevalence in rural in comparison to peri-urban areas was further depicted by comparing the dose–response curves between these areas.

**Figure 4:**
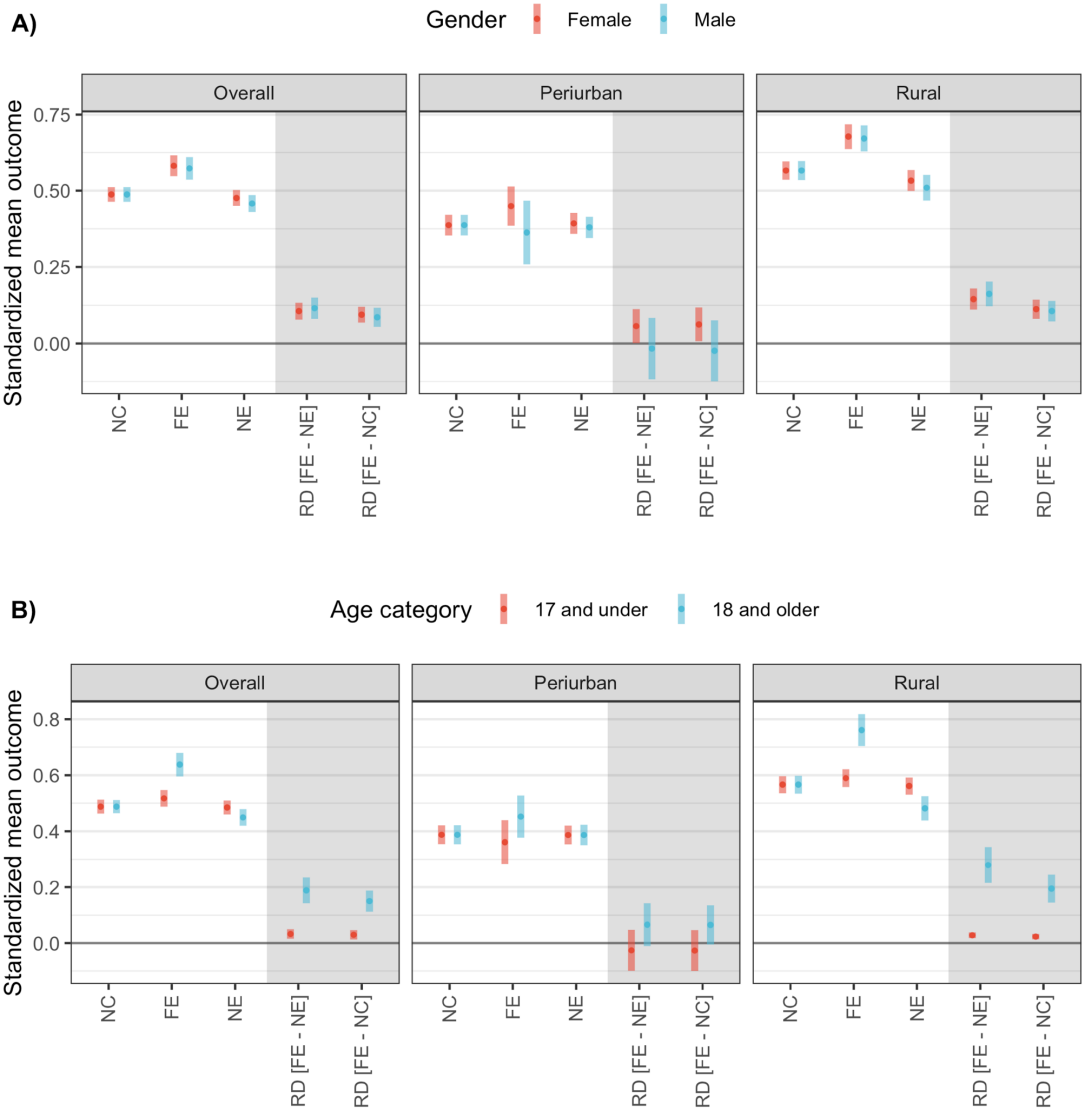
Standardized mean outcome estimated under simulated scenarios by geographic area and (**A**) gender and (**B**) age in the Loreto department in the Peruvian Amazon. Standardized mean outcome estimations for the natural course (NC) and simulated scenarios of full exposure (FE), no exposure (NE) and their risk differences (RD, grey area).

Further scenarios were explored based on gender and age travel restriction policies. The standardized mean outcome when simulating a full exposure (FE–100% prevalence of out-of-village working activities) was 57.3% (95% CI: 53.6%–61.0%) in males and 58.2% (95% CI: 54.8%–61.6%) in females (Fig. 4A). In contrast, the standardized mean outcome when simulating the NE was 45.8% (95% CI: 43.0%–48.6%) and 47.6% (95% CI: 45.0%–50.2%) in males and females, respectively. Overall, a slightly greater impact was observed when restriction policies targeted males. The average causal effect in males was 11.5% (95% CI: 8.0%–15.0%) and 10.5% (95% CI: 7.8%–13.3%) in females. Importantly, this type of policy is most effective in rural areas (Fig. 4A). The average causal effect is 3- and 15-folds higher in rural than peri-urban areas, in females and males respectively.

A contrasting pattern was observed when simulating travel restrictions based on legal adult age (18 years old) in Peru. The standardized mean outcome when simulating the FE was greater in adults—18 years old and older—(63.8%; 95% CI: 59.6%–68.0%) than in children and adolescents—17 years old and under—(51.7%; 95% CI: 48.7%–54.7%) and (Fig. 4B). In addition, a pronounced reduction in the standardized mean outcome was observed in adults (44.9%; 95% CI: 42.0%–47.9%) in comparison to children and adolescents (48.5%; 95% CI: 46.0%–50.9%) when simulating the NE. Overall, a greater impact was observed when restriction policies were targeted at adults than children and adolescents. The average causal effect in adults was 18.8% (95% CI: 14.2%–23.5%) and 3.2% (95% CI: 1.5%–5.0%) in children and adolescents. As previously observed, this policy scenario (targeting mobility restrictions to adults) is only effective in rural than peri-urban areas (Fig. 4B). In rural areas, we identified an average causal effect of 27.9% (95% CI: 21.5%–34.3%) while no effect in peri-urban areas.

## Discussion

Despite numerous studies highlighting the links between human mobility and malaria in Amazonian contexts, the quantification of occupational-driven mobility was lacking. Using two population-based studies we determined the average causal effect of out-of-village working activities on malaria seropositivity (recent exposure to the malaria parasite). This study highlighted the critical role of human population mobility in sustaining malaria transmission in the Peruvian Amazon. By simulating the prevalence of out-of-village working activities to reflect different policy scenarios, this study showed the importance of targeting key subpopulations when designing such occupational interventions. Particularly, targeting males and adult (18 years old and older) populations causes the greatest effect on malaria seropositivity. Finally, in all these scenarios, the effect is highly pronounced in rural in comparison to peri-urban areas. The findings of this study are substantial to tailor current and future malaria elimination programs in the Amazon Region.

The role of human population mobility is of particular importance under elimination and prevention of reintroduction frameworks^9,10,41^. In areas where malaria transmission is heterogeneous—such as the Peruvian Amazon—, human mobility increases the importation risk (formerly known as vulnerability)^28^. Multiple mechanisms originate different mobility patterns^42–45^ as described in Africa^46,47^, Southeast Asia^48^, and more recently in Latin America^14,49^. Out-of-village working activities are central in the Peruvian Amazon since it is the most frequent reason for human mobility^13^. However, as previously described^42,50–52^, subnational and local approaches should be considered since human mobility and malaria dynamics are tightly related micro-geographical and local contexts. In this study, we estimated the contrasting effect of a set of policy scenarios in rural and peri-urban areas. Importantly, both settings are located in contiguous districts (administrative level 3). This emphasizes the importance to adopt flexible malaria elimination approaches since a variety of scenarios could be found at neighbor subnational levels.

The goal of this study was to simulate the benefits of a new set of interventions by focusing solely on hypothetical interventions linked to out-of-village working activities. We recommend that future directions in malaria elimination research and policy would quantify other historical interventions based on pharmacological, environmental, and social/lifestyle factors to define a cost-effective set of interventions to achieve local malaria elimination goals. The hypothetical set of interventions tested in this study does not intend to suggest limiting the mobility of habitants in the Amazon Region, rather, intends to highlight their key role in sustaining malaria transmission. In consequence, our findings emphasized the urge to design tailored interventions for subpopulations that contribute the most to the malaria exposure such as males and adults in rural areas. Based on previous experiences^5^, community health workers (CHW) may play a key role in deploying such kind of targeted strategies.

This study concludes that out-of-village working activities potentially encompass a wide range of mobility patterns that, if correctly identified, may help to enhance targeted interventions such as screening or surveillance strategies. Furthermore, recent studies leverage detailed GPS data to identify where people go and spend their time, allowing them to obtain accurate measurements of what they are exposed to within their activity spaces^53^. Studies in Southeast Asia^48^ and Latin America^14^ showed the interaction between travel/commuting patterns and land coverage as a main driver of malaria endemicity. Importantly, *Ny. darlingi* (dominant malaria vector) demonstrates an increased exophagic–outdoors–biting behavior^29,54,55^ and a breeding site preference in the forest fringes^56–58^ in rural Amazon. Taken together, if both environmental and health policies are combined, it is hypothesized that amplified impacts in both fields can be achieved^59^.

We acknowledge the following limitations in this study. First, as a cross-sectional, this study is not designed to infer malaria transmission intensity. In this case, the seroprevalence reflects recent exposure to malaria infection (up to 9 months in the past)^31,33^, however no active infection data was used. Despite other studies demonstrating that malaria seroprevalence is a good proxy for malaria transmission intensity^11,60,61^, further longitudinal studies, to deal with potential regressions to the mean issues, are suggested to determine the causal effect on malaria transmission intensity. Second, personal protective measures (i.e., seasonal mobility, the use of bed nets or pharmacological prophylaxis) may play a key role in effect modification. Furthermore, besides the inclusion of these behaviors, considering the timing during transit or return of out-of-village activities may be important to consider for future studies. Finally, a potential threat to causal identifiability in this study may arise from the fact the outcome (secondary infection) in one individual may be dependent on the outcome (primary infection) in other individuals (in other words, it would violate the stable unit treatment value assumption)^62^. Given the design of this study, the main assumption relies on that the main outcome (recent malaria exposure) is independent across study participants (i.e., no interference). Further longitudinal studies including GPS data may explicitly determine the interactions (matrices) between primary and secondary infections to estimate causal effects that are conditional on contact with an exposed individual^63,64^.

## Conclusion

The findings of this study support the hypothesis that targeting out-of-village workers will considerably benefit current malaria elimination strategies in the Amazon Region. Particularly, males and adult populations that carried out out-of-village working activities in rural areas contribute the most to the malaria seropositivity (recent exposure to the parasite) in the Peruvian Amazon. This study contributed to designing a new set of interventions that will potentially prevent one-third of recent malaria exposures. An optimal set of interventions to achieve malaria elimination goals in Amazonia should be driven by further exploring the causal effects of innovative and traditional policies.

## Supplementary Information

Supplementary Information.

## Data Availability

Data used in this study is available at https://doi.org/10.6084/m9.figshare.19802116.v1.

## Code availability

Code used in this study is available at https://bookdown.org/gabc91/ch3_hpm_gcomp/.
