# Supplementary material for: Effect of out-of-village working activities on recent malaria exposure in the Peruvian Amazon using parametric g-formula
Source: Sci Rep. 2022 Nov 9;12:19144. doi: 10.1038/s41598-022-23528-8 (PMC9645738; doi:10.1038/s41598-022-23528-8)

Effect of out-of-village working activities on recent malaria exposure

in the Peruvian Amazon using parametric g-formula

Gabriel Carrasco-Escobar^1,2,*^, Jason Rosado^3^, Oscar Nolasco^4,5^, Michael T. White^3^, Ivo Mueller^6,7^, Marcia C. Castro^8^, Hugo Rodriguez-Ferruci^9^, Dionicia Gamboa^4,5,10^, Alejandro Llanos-Cuentas^4^, Joseph M. Vinetz^4,5,11^, Tarik Benmarhnia^12^

**Affiliations:**

^1^ Herbert Wertheim School of Public Health and Human Longevity Science, University of California San Diego, La Jolla, CA, USA

^2^ Health Innovation Lab, Institute of Tropical Medicine “Alexander von Humboldt”, Universidad Peruana Cayetano Heredia, Lima, Peru

^3^ G5 Épidémiologie et Analyse des Maladies Infectieuses, Département de Santé Globale, Institut Pasteur, F-75015, Paris, France

^4^ Instituto de Medicina Tropical Alexander von Humboldt, Universidad Peruana Cayetano Heredia, Lima, Peru

^5^ Laboratorio ICEMR-Amazonia, Laboratorios de Investigación y Desarrollo, Facultad de Ciencias y Filosofía, Universidad Peruana Cayetano Heredia, Lima, Peru

^6^ Department of Medical Biology, University of Melbourne, Australia

^7^ Population Health and Immunity Division, Walter and Eliza Hall Institute of Medical Research, Melbourne, Australia

^8^ Department of Global Health and Population, Harvard T.H. Chan School of Public Health, Boston, MA, United States of America

^9^Universidad Nacional de la Amazonía Peruana, Loreto, Peru

^10^Departamento de Ciencias Celulares y Moleculares, Facultad de Ciencias y Filosofía, Universidad Peruana Cayetano Heredia, Lima, Peru

^11^ Section of Infectious Diseases, Department of Internal Medicine, Yale School of Medicine, New Haven, CT, USA

^12^ Scripps Institution of Oceanography, University of California, San Diego, CA 92037

*** Correspondence:**Gabriel Carrasco-Escobar, MSc, PhD(c): [gabriel.carrasco@upch.pe](mailto:gabriel.carrasco@upch.pe)

**Email contacts:**

Jason Rosado: [javier.rosado@pasteur.fr](mailto:javier.rosado@pasteur.fr)

Oscar Nolasco: [oscar.nolasco.c@upch.pe](mailto:oscar.nolasco.c@upch.pe)

Michael T. White: [michael.white@pasteur.fr](mailto:michael.white@pasteur.fr)

Ivo Mueller: [mueller@wehi.edu.au](mailto:mueller@wehi.edu.au)

Marcia C. Castro: [mcastro@hsph.harvard.edu](mailto:mcastro@hsph.harvard.edu)

Hugo Rodriguez-Ferruci: [hmrodriguezf@hotmail.com](mailto:hmrodriguezf@hotmail.com)

Dionicia Gamboa: [dionigamboa@yahoo.com](mailto:dionigamboa@yahoo.com)

Alejandro Llanos-Cuentas: [alejandro.llanos.c@upch.pe](mailto:alejandro.llanos.c@upch.pe)

Joseph M. Vinetz: [joseph.vinetz@yale.edu](mailto:joseph.vinetz@yale.edu)

Tarik Benmarhnia: [tbenmarhnia@health.ucsd.edu](mailto:tbenmarhnia@health.ucsd.edu)

**SUPPLEMENTARY INFORMATION**

**Supplementary Figure 1. Household seropositivity rate spatial distribution in villages in the study area in the Loreto department in the Peruvian Amazon.** Maps were produced using R v.4.1 (R Development Core Team, R Foundation for Statistical Computing, Australia) based on public geographic data extracted from OpenStreetMap contributors ([www.openstreetmap.org](http://www.openstreetmap.org)) under Open Data Commons Open Database License (ODbL) 1.0 (<http://openstreetmap.org/copyright>).

**
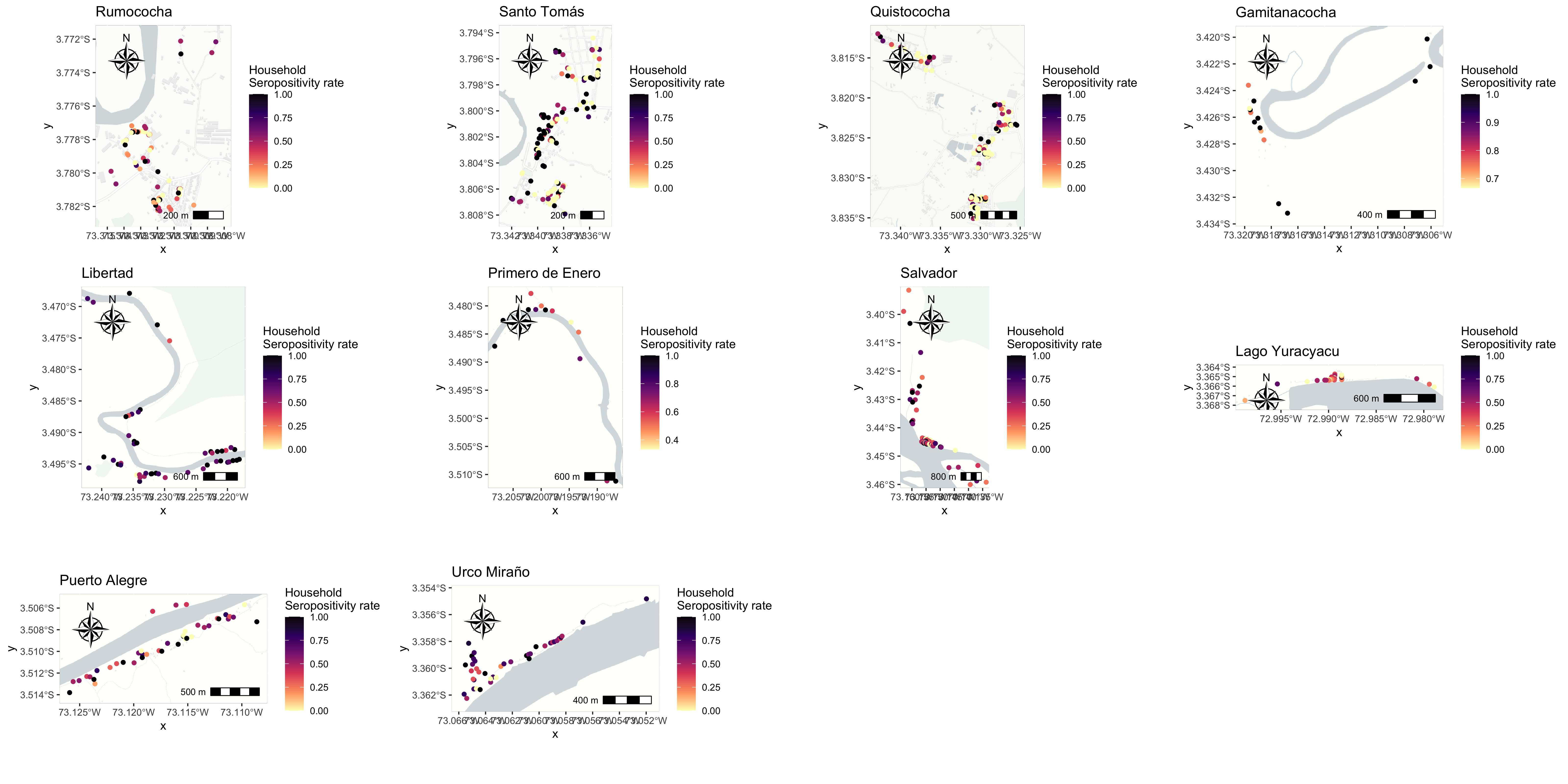
**

**Supplementary Figure 2. Frecuency of main occupational activity grouped by out-of-village and in-village activities and seropositivity status.**

**
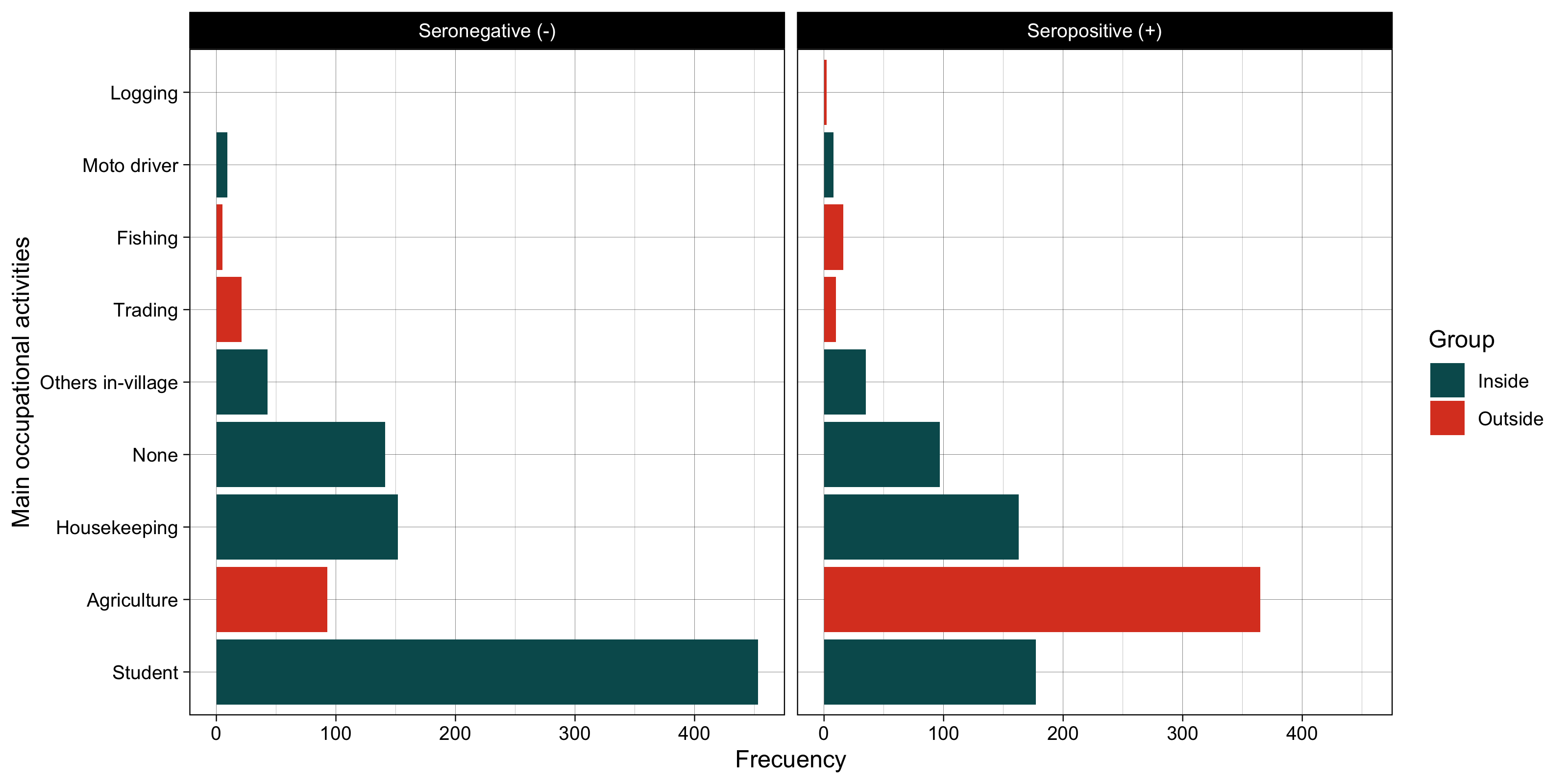
**

**Supplementary Figure 3. Household work out-of-village rate spatial distribution in villages in the study area in the Loreto department in the Peruvian Amazon.** Maps were produced using R v.4.1 (R Development Core Team, R Foundation for Statistical Computing, Australia) based on public geographic data extracted from OpenStreetMap contributors ([www.openstreetmap.org](http://www.openstreetmap.org)) under Open Data Commons Open Database License (ODbL) 1.0 (<http://openstreetmap.org/copyright>).


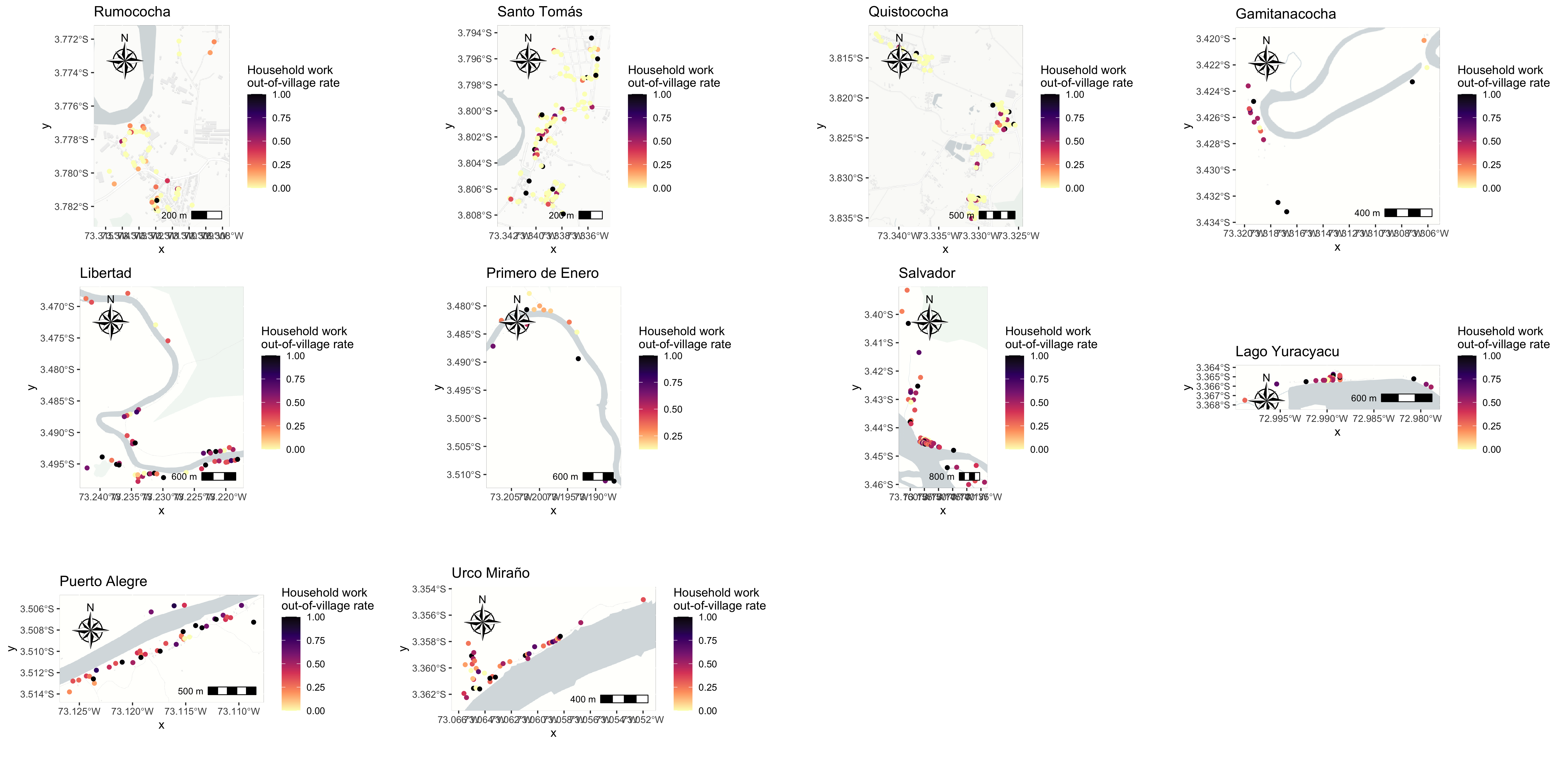

Supplement: Supplementary file 1 — Supplementary Information. [file 41598_2022_23528_MOESM1_ESM.docx]
